# Serum 25-hydroxyvitamin D3 concentration in Iranian patients with Parkinson's disease

**Published:** 2013

**Authors:** Mehdi Moghaddasi, Mansoureh Mamarabadi, Mahboubeh Aghaii

**Affiliations:** Department of Neurology, Tehran University of Medical Sciences, Tehran, Iran

**Keywords:** Vitamin D3, Parkinson's Disease, Postural Instability

## Abstract

**Background:**

Vitamin D is an important factor responsible for many physiologic functions. Vitamin D deficiency is associated with increased risk of neurodegenerative disease. The level of vitamin D in Iranian patients with Parkinson's disease and its relationship with severity of symptoms and signs were evaluated in this study.

**Methods:**

Eighty-three patients with Parkinson's disease (PD) were recruited using simple non-random sampling. 25-hydroxyvitamin D [25(OH)D3] was measured by Electrochemiluminescence immunoassay (ECLIA). Serum level of calcium and phosphorus was measured to exclude other endocrine disorders.

**Results:**

The mean 25(OH)D3 concentration was lower in the PD population than in the normal group. Lower levels of 25(OH)D3 were associated with more severe postural instability and abnormal posture. There was no significant association between levels of 25(OH)D3 and severity of other symptoms of parkinsonism.

**Conclusion:**

This analysis showed that serum 25(OH)D levels are lower in PD patients in comparison with normal range. In addition, there was a significant association between the presences of freezing, postural instability and abnormal postures with lower levels of 25(OH)D.

## Introduction

Vitamin D has an important role in many physiologic functions; moreover, vitamin D deficiency is associated with increased risk of several types of cancers, as well as autoimmune and cardiovascular disorders.^1–3^ Vitamin D functions include perseveration of mineral bone density, muscle strength and innate immunity.^1, 2^

Indeed, vitamin D is necessary for regulation of some neurodegenerative processes such as neurotrophin, inducible nitric oxide synthase (iNOS), glutathione and monoamine synthesis and also apoptosis.^4, 5^ These mechanisms are probable pathogenesis of some neurodegenerative diseases such as multiple sclerosis (MS) and Parkinson's disease (PD).^6^

Given the expression of both 1-hydroxylase (1-OHase) and vitamin receptors (VDRs) in many extra renal tissues -including muscle and brain -vitamin D status may be important for prevention or treating neurodegenerative disorders.^7, 8^

In brain, hippocampus and substantia nigra neurons demonstrate high concentrations of VDRs in their nucleus and 1-OHase in their cytosol. Some previous investigations concerning vitamin D deficiency in PD focused on skeletal health associations in old Asian populations.^9–12^ Besides, epidemiologic, animal, and human data supported that vitamin D deficiency may be involved in the pathogenesis, progression, and clinical manifestations of PD.^6^

Parkinson's disease (PD) is a movement disorder and is one of the most common causes of disability in the elderly. This neurodegenerative disease is characterized by tremor, rigidity, hypokinesia, bradykinesia, and loss of postural reflexes that leads to immobility and frequent falls.^13, 14^

Some studies demonstrated that deficiency of 25-hydroxyvitamin D [25(OH)D] and immobilization contribute to reduced bone mineral density (BMD) in PD patients, recurrent falling, lumbosacral and hip fractures.^9, 15–17^ In this study, the association of serum vitamin D concentration and PD symptoms severity were reviewed.

## Materials and Methods

The study was approved by the Medical Ethics Committee of Tehran University of Medical Sciences.

### Patients

Eighty-three PD subjects, who referred to Neurology Clinic of Rasoul-e-Akram Hospital (affiliated to Tehran University of Medical Sciences, Iran), were randomly recruited in the study. Informed consent was obtained from all the subjects before the intervention. Diagnosis of PD was confirmed with comorbidity of abnormal movement disorders.

The patients filled out a questionnaire about their demographic characteristics (age, sex, etc.), duration of illness, initial symptom, comorbidity and last time of medication consumption. Physical examination was done by a neurologist; the scores are shown in Table 1.

**Table 1:** Scoring Parkinson's Disease signs in physical examination

Signs	Score
Tremor at rest	0 = Absent 1 = Slight and infrequently present 2 = Mild in amplitude and persistent, or moderate in amplitude but only intermittently present 3 = Moderate in amplitude and present most of the time 4 = Marked in amplitude and present most of the time
Hypokinesia and Bradykinesia	0 = None 1 = Minimal slowness, giving movement a delibe rate character; could be normal l for some people; possibly reduced amplitude 2 = Mild degree of slowness, giving poverty of movement that is definitely abnormal; alternatively, some reduced amplitude 3 = Moderate slowness; poverty or small amplitude of movement 4 = Marked slowness; poverty or small amplitude of movement
Rigidity	0 = Absent 1 = Slight or detectable only when activated by mirror or other movements 2 =Mild to moderate 3 = Marked, but full range of motion easily achieved 4 = Severe; range of motion achieved with difficulty
Posture	0 = Normal 1 = Not quite erect, slightly stooped posture; could be normal l for older person 2 = Moderately stooped posture, definitely abnormal l; can be slightly leaning to one side 3 = Severely stooped posture with kyphosis; can be moderately leaning to one side 4 = Marked flexion with extreme abnormality of posture
Postural stability	0 = Normal 1 = Retropulsion, but recovers unaided 2 = Absence of postural response; would fall if not caught by examiner 3 = Very unstable; tends to lose balance spontaneously 4 = Unable to stand without assistance
freezing	0 = None 1 = Rare freezing when walking; may have start hesitation 2 = Occasional freezing when walking 3 = Frequent freezing; occasionally falls from chills 4 = Frequent falls from freezing

### Laboratory Tests

Blood samples (4cc) were taken through an intravenous cannula inside the forearm and were transferred to the laboratory in cold boxes.

Serums were analyzed for 25(OH)D3 concentration with Electrochemiluminescence immunoassay (ECLIA). Serum level of calcium, phosphorus and PTH were also assessed for exclusion of calcium and phosphorus metabolism disorders. Patients with history of stroke, transient ischaemic attack (TIA), essential tremor (ET), restless leg syndrome, CRF and hypo-hyperparathyroidism and those who underwent deep brain stimulation (DBS) surgeries were excluded from the study.

### Statistical Analysis

Data were analyzed using Student's t-test, paired t-test and one-way analysis of variance (ANOVA). The Spearman correlation analysis test was performed for correlative studies. Significance for all tests was set at P = 0.05. All the statistical analyses were performed with SPSS for windows 16.0 (SPSS Inc., Chicago, IL, USA).

## Results

In this study, 83 patients were studied. Mean age of the patients was 56.57 ± 11.71 years (age range 24-79 years); 63 of them (75.9%) were males and 20 were females (24.1%). Mean age of symptoms onset was 50.71 ± 12.10 years (range 20-77 years).

Seasons of sampling were variable with the most sampling in summer (32 patients; 38.6%), blood sampling of 28 patients (33.7%) was in fall and 23 patients (27.7%) in spring. Resting tremor with frequency of 62.7% (52 patients) was the most common initial symptom of the disease.

Hypokinesia-bradykinesia 36.1% (30 patients), and rigidity 1.2% (1 patient) were other initial symptoms. The mean 25(OH)D level was 17.60 ± 16.89 ng/ml (range 4-98 ng/ml) (normal range of 16-40 ng/ml). There was a significant difference between 25(OH)D of PD patients compared to the normal range of 25(OH)D (P < 0.01) and levels of 25(OH)D according to the seasons of sampling.

25(OH)D levels were significantly higher in spring in comparison with summer and fall seasons (P < 0.01). There was no significant changes between 25(OH)D levels in summer sampling and fall sampling (P = 0.60) (Table 2).

**Table 2:** 25(OH)D levels in different seasons for blood sampling

Season	25(OH)D levels/mean ± SD (range with 95% CI) (ng/ml)
Spring	35.43 ± 18.90 (27.25-43.61)
Summer	11.89 ± 11.22 (7.84-15.93)
Fall	9.4 ± 7.28 (6.65-12.30)

There was no significant difference in 25(OH)D levels between males and females (17.82 ± 17.85 ng/ml vs. 16.91 ± 13.84 ng/ml) (P = 0.93). 25(OH)D levels were increased with age increase (r = 0.2).

There was a significant difference between 25(OH)D levels in patients younger than 50 years compared to the older ones (10.92 ± 11.03 vs. 20.16 ± 18.09 ng/ml) (P = 0.02). There was a reverse association between duration of disease and 25(OH)D levels (r = -0.1).

Our analysis showed no significant association between 25(OH)D levels and initial symptom of the disease (P = 0.28), severity of resting tremor (P = 0.5), bradykinesia/hypokinesia (P = 0.10) and chills (P = 0.7).

There was a major association between 25(OH)D levels and severity of postural instability (P = 0.05), impaired posture (P = 0.02) and presence or absence of freezing(14.01 ± 14.09 vs. 25.48 ± 19.94 ng /ml) (P < 0.01) (Table 3).

**Table 3:** Level of 25(OH)D in relevant signs

Signs	Presence of signs	25(OH)D levels (ng/ml)
Postural stability	Yes	21.09 ± 19.31
	No	13.26 ± 12.21
Freezing	Yes	14.01 ± 14.09
	No	25.48 ± 19.94

## Discussion

Vitamin D deficiency is an important condition in the elderly. Prevalence of neurodegenerative disease is also higher in these patients. Vitamin D is produced in body in skin on exposure to UV-B radiation and is found in limited food sources.^1, 18^

Some involved factors in vitamin D deficiency are advanced age, obesity, avoidance of sun exposure, residence in northerly latitudes, and darker skin. Patients with PD have many risk factors for vitamin D deficiency such as advanced age, immobility and activation due to chills or abnormal posture and malnutrition. Serum 25(OH)D is the most useful indicator of vitamin D level of body.^19^

It is derived from both dietary intake and sunlight-induced production by the skin.^20^ In our study, mean 25(OH)D level was lower in comparison with previous studies. In the study of Evatt et al.^21^ in Atlanta, the mean 25(OH)D levels was 31.9 ± 13.6 which was significantly higher than our patients. It is probably due to differences between geographical latitude, sun radiation, coating conditions and amount of skin pigmentation.

In our study, results suggested that serum 25(OH)D levels were lower in PD patients in comparison with normal range. Furthermore, there was a significant association between the presence of chills, postural instability and abnormal postures with lower levels of 25(OH)D. These results may be due to sunlight deprivation due to immobilization, together with decreased dietary intake of vitamin D.

In the study of Sato et al., the higher incidence of nonvertebral fractures was seen in female patients with PD that might be due to frequent falls and osteoporosis caused by deficiency of vitamins D.^22^ Supplementation of vitamin D and increased sun exposure may improve the quality of life of patients with Parkinson's disease and decrease complications such as falling and fractures.

Sato et al. examined elderly patients who were immobilized chronically from PD to assess the effect of sun shining in the development of hip and vertebral fractures.^23^ The high incidence of hip fractures in elderly PD patients might be attributed to the frequent falls and osteoporosis due to vitamin D deficiency. A recent study suggested that vitamin D supplementation reduced the risk of falls among ambulatory or institutionalized older individuals.^23^

In conclusion, this analysis showed that serum 25(OH)D levels were lower in PD patients in comparison with normal range. Moreover, there was a significant associations between the presence of chills, postural instability and abnormal postures with lower levels of 25(OH)D.

Limitations of the present study were lack of controlling dietary intake and exposure to sunlight, and also the portion of plasma samples drawn in the spring and fall to summer were not matched with each other.

## References

[1] Holick MF (2007). Vitamin D deficiency. N Engl J Med.

[2] Bischoff-Ferrari HA, Dietrich T, Orav EJ, Hu FB, Zhang Y, Karlson EW (2004). Higher 25-hydroxyvitamin D concentrations are associated with better lower-extremity function in both active and inactive persons aged > or =60 y. Am J Clin Nutr.

[3] Zittermann A (2006). Vitamin D and disease prevention with special reference to cardiovascular disease. Prog Biophys Mol Biol.

[4] Garcion E, Wion-Barbot N, Montero-Menei CN, Berger F, Wion D (2002). New clues about vitamin D functions in the nervous system. Trends Endocrinol Metab.

[5] Baksi SN, Hughes MJ (1982). Chronic vitamin D deficiency in the weanling rat alters catecholamine metabolism in the cortex. Brain Res.

[6] Newmark HL, Newmark J (2007). Vitamin D and Parkinson's disease--a hypothesis. Mov Disord.

[7] Eyles DW, Smith S, Kinobe R, Hewison M, McGrath JJ (2005). Distribution of the vitamin D receptor and 1 alpha-hydroxylase in human brain. J Chem Neuroanat.

[8] Holick MF (2004). Vitamin D: importance in the prevention of cancers, type 1 diabetes, heart disease, and osteoporosis. Am J Clin Nutr.

[9] Sato Y, Kikuyama M, Oizumi K (1997). High prevalence of vitamin D deficiency and reduced bone mass in Parkinson's disease. Neurology.

[10] Sato Y, Honda Y, Iwamoto J, Kanoko T, Satoh K (2005). Abnormal bone and calcium metabolism in immobilized Parkinson's disease patients. Mov Disord.

[11] Sato Y, Iwamoto J, Kanoko T, Satoh K (2006). Alendronate and vitamin D2 for prevention of hip fracture in Parkinson's disease: a randomized controlled trial. Mov Disord.

[12] Vaserman N (2005). Parkinson's disease and osteoporosis. Joint Bone Spine.

[13] Aita JF (1982). Why patients with Parkinson's disease fall. JAMA.

[14] Koller WC, Glatt S, Vetere-Overfield B, Hassanein R (1989). Falls and Parkinson's disease. Clin Neuropharmacol.

[15] Chiu KY, Pun WK, Luk KD, Chow SP (1992). Sequential fractures of both hips in elderly patients--a prospective study. J Trauma.

[16] Grisso JA, Kelsey JL, Strom BL, Chiu GY, Maislin G, O'Brien LA (1991). Risk factors for falls as a cause of hip fracture in women. The Northeast Hip Fracture Study Group. N Engl J Med.

[17] Johnell O, Melton LJ, Atkinson EJ, O'Fallon WM, Kurland LT (1992). Fracture risk in patients with parkinsonism: a population-based study in Olmsted County, Minnesota. Age Ageing.

[18] Lawson DE, Paul AA, Black AE, Cole TJ, Mandal AR, Davie M (1979). Relative contributions of diet and sunlight to vitamin D state in the elderly. Br Med J.

[19] Nieves J, Cosman F, Herbert J, Shen V, Lindsay R (1994). High prevalence of vitamin D deficiency and reduced bone mass in multiple sclerosis. Neurology.

[20] Beadle PC (1977). Sunlight, ozone and vitamin D. Br J Dermatol.

[21] Evatt ML, Delong MR, Khazai N, Rosen A, Triche S, Tangpricha V (2008). Prevalence of vitamin d insufficiency in patients with Parkinson disease and Alzheimer disease. Arch Neurol.

[22] Sato Y, Honda Y, Kaji M, Asoh T, Hosokawa K, Kondo I (2002). Amelioration of osteoporosis by menatetrenone in elderly female Parkinson's disease patients with vitamin D deficiency. Bone.

[23] Sato Y, Iwamoto J, Honda Y (2011). Amelioration of osteoporosis and hypovitaminosis D by sunlight exposure in Parkinson's disease. Parkinsonism Relat Disord.

